# Dalbavancin: novel candidate for COVID-19 treatment

**DOI:** 10.1038/s41422-020-00459-5

**Published:** 2021-01-20

**Authors:** Markus Hoffmann, Yeonhwa Jin, Stefan Pöhlmann

**Affiliations:** 1 Infection Biology Unit, German Primate Center, 37077 Göttingen, Germany; 2 Faculty of Biology and Psychology, Georg-August-University Göttingen, 37073 Göttingen, Germany

**Keywords:** Immunology, Molecular biology

**Antivirals approved for treatment of severe acute respiratory syndrome coronavirus 2 (SARS-CoV-2) infection and the associated coronavirus disease 2019 (COVID-19) are largely lacking. In a recent study in** ***Cell Research***, **Wang et al. show that the clinically proven antibiotic dalbavancin blocks SARS-CoV-2 binding to its receptor ACE2 and reduces viral spread and pathogenesis in animal models**.

The coronavirus disease 2019 (COVID-19) pandemic has caused a high global disease burden and death toll, dramatic economic losses, and ensuing social hardships. More than 2000 patients are currently dying from COVID-19 every day in the US alone and COVID-19-associated deaths have globally surpassed one million. Despite intensive efforts to identify new drugs or to repurpose existing drugs for COVID-19 treatment, only few promising candidates are available. Remdesivir, an inhibitor of the viral polymerase, has been reported to reduce time of hospitalization,^1^ but recent studies questioned the clinical usefulness of this drug.^2^ Dexamethasone, which diminishes the uncontrolled expression of cytokines during the hyperinflammation phase of COVID-19, was shown to reduce COVID-19-associated mortality^3^ but does not exert antiviral activity. Initial data indicate that RNA- as well as vector-based vaccines might be effective. However, a notable impact of vaccines on the course of the pandemic is only expected for the second half of 2021 and a significant fraction of the population might not have access to vaccines or might opt not to use them. Therefore, potent antivirals are urgently needed.

Severe acute respiratory syndrome coronavirus 2 (SARS-CoV-2) and its cousin, SARS-CoV, which was responsible for the SARS epidemic in 2002/2003, use the cellular carboxypeptidase angiotensin-converting enzyme 2 (ACE2) as receptor for entry.^4,5^ Binding of the viral spike protein (S) to ACE2 is essential for SARS-CoV and SARS-CoV-2 entry into target cells, although engaging other cellular factors like heparan sulfate proteoglycans can increase ACE2-dependent entry. The interface between ACE2 and the S proteins of SARS-CoV and SARS-CoV-2 has been elucidated at the atomic level. Nevertheless, strategies to target this interface for antiviral intervention are limited, with the exception of antibodies directed against the receptor binding domain (RBD) of the viral S proteins and the development of soluble ACE2 for SARS and COVID-19 therapy.^6,7^

**Fig. 1:**
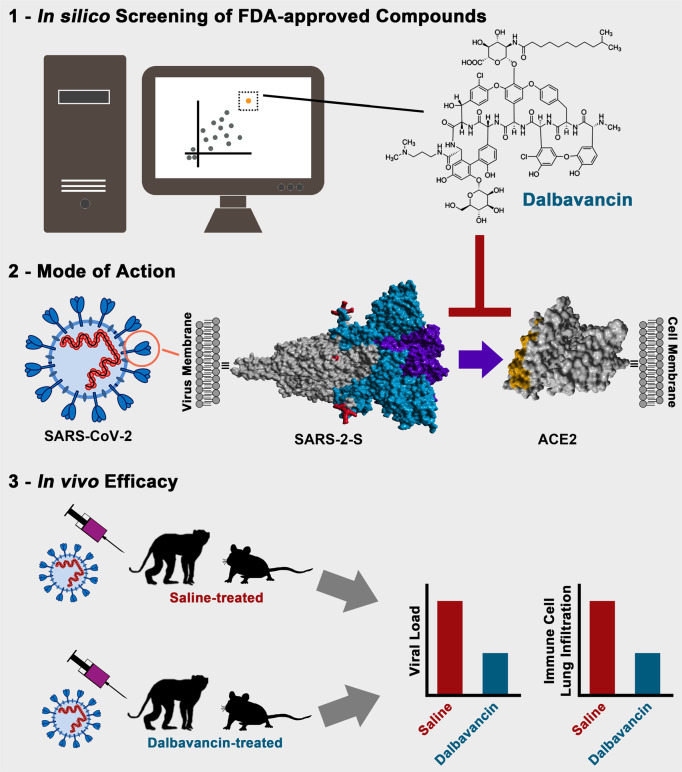
Identification, mode of action and in vivo efficacy of dalbavancin—a novel candidate for COVID-19 treatment. Dalbavancin was identified in an in silico screen for compounds that bind to ACE2, the receptor used by SARS-CoV and SARS-CoV-2 for entry into cells. Dalbavancin binds to ACE2 and blocks SARS-CoV-2 entry in cell culture. Finally, dalbavancin reduces SARS-CoV-2 replication and disease development in animal models.

A timely and comprehensive analysis by Wang et al. now shows that the antibiotic dalbavancin blocks the interaction of S protein with ACE2 and inhibits viral spread.^8^ They identified dalbavancin in an in silico screen of FDA-approved compounds for ACE2 binders and demonstrate that dalbavancin interacts with ACE2 and blocks SARS-CoV-2 S (SARS-2-S) binding to ACE2 (Fig. 1). Moreover, dalbavancin is shown to inhibit SARS-2-S-driven entry into target cells and SARS-CoV-2 infection of cultured cells. Finally, dalbavancin treatment protects experimentally infected mice and macaques from lung lesions, infiltration of inflammatory cells and dysregulated expression of proinflammatory cytokines associated with the hyperinflammation phase of COVID-19 (Fig. 1). Thus, dalbavancin, a glycopeptide antibiotic approved for treatment of acute bacterial skin and skin structure infections, might constitute a novel treatment option for COVID-19.

Dalbavancin is not a novel candidate in the arena of coronavirus therapeutics. A previous study showed that dalbavancin blocks cell entry driven by SARS-CoV S (SARS-S) and the Ebola virus glycoprotein (EBOV-GP).^9^ Moreover, the study demonstrated that dalbavancin inhibits the cellular cysteine protease cathepsin L, which activates SARS-S and EBOV-GP. Wang and colleagues propose a minor contribution of cathepsin L inhibition to dalbavancin-mediated blockade of SARS-CoV-2 infection. However, further analyses are required to define the exact contribution of cathepsin L blockade to antiviral activity of dalbavancin, since one cell line used to address this question, the colorectal adenocarcinoma-derived cell line Caco-2, expresses both cathepsin L and another S protein-activating protease, the cellular serine protease TMPRSS2, and may allow for S protein activation by both proteases.^10^ Addressing this question is important considering that TMPRSS2 but not cathepsin L is believed to activate SARS-S and SARS-2-S in lung tissue.^11^ As a consequence, any dalbavancin-mediated, cathepsin L-dependent blockade of SARS-CoV-2 in cell culture might not translate into antiviral activity in the infected host, although viral spread in heart and potentially other extrapulmonary organs could be TMPRSS2-independent.

A major strength of the present study is the clear demonstration that dalbavancin blocks SARS-2–S interaction with ACE2 as well as the identification of ACE2 residues critical for dalbavancin binding. Importantly, the RBD is structurally highly conserved between SARS-S and SARS-2-S and both S proteins bind to the same region on ACE2. Dalbavancin binding to ACE2 should thus inhibit both viruses as well as closely-related viruses detected in bats, several of which bind ACE2 and might have zoonotic potential. Dalbavancin would thus join the list of compounds that block host cell factors essential for spread of emerging coronaviruses and could be used to combat future outbreaks. Whether dalbavancin also inhibits ACE2 engagement by the S protein of the human coronavirus NL63, which can cause severe lung disease in rare cases, remains to be investigated. Another strength of the study is the demonstration of dalbavancin’s antiviral activity in both mice transgenic for human ACE2 and rhesus macaques (Fig. 1). In both models, dalbavancin markedly reduced development of pneumonia and lung lesions. Moreover, dalbavancin protected mice from body weight loss and reduced the expression of IL-8 and MCP-1 in lung tissues of macaques, although expression of other cytokines, including IL-6, was not significantly altered.

In sum, dalbavancin is a clinically proven antibiotic that could be repurposed for COVID-19 treatment and further evaluation is called for. Future challenges might include the route of application—dalbavancin is applied intravenously—and the question of whether SARS-CoV-2 can acquire resistance.
