# Publisher Correction: Dalbavancin: novel candidate for COVID-19 treatment

**DOI:** 10.1038/s41422-022-00713-y

**Published:** 2022-09-01

**Authors:** Markus Hoffmann, Yeonhwa Jin, Stefan Pöhlmann

**Affiliations:** 1 Infection Biology Unit, German Primate Center, 37077 Göttingen, Germany; 2 Faculty of Biology and Psychology, Georg-August-University Göttingen, 37073 Göttingen, Germany

**Keywords:** Immunology, Molecular biology

Correction to: *Cell Research* 10.1038/s41422-020-00459-5, published online 20 January 2021

The licence information was missing from this article and should have been CC-BY.

The original article has been corrected.

